# Editorial: Science of science: a complex network perspective

**DOI:** 10.3389/frma.2025.1595966

**Published:** 2025-04-10

**Authors:** Sandro M. Reia, Filipi N. Silva, Henrique F. de Arruda

**Affiliations:** ^1^Geography and Geoinformation Science, College of Science, George Mason University, Fairfax, VA, United States; ^2^Observatory on Social Media, Indiana University, Bloomington, IN, United States

**Keywords:** network science, complex networks, visualization, applied science, network structure

Science of science (SciSci) is the field that studies the mechanisms underlying science (Fortunato et al., 2018). SciSci has been shown to be essential for the development of science as a whole, with potential implications for research efficiency, understanding career trajectories, and accelerating innovation and collaboration patterns (e.g., enabling better mentoring and funding allocation). Network science has emerged as a powerful tool within SciSci, enabling the analysis and modeling of the scientific ecosystem through various types of networks, such as citation and collaboration networks. In this context, the Research Topic “*Science of science: a complex network perspective*” is timely and has captured different applications of network science in SciSci.

Valejo et al. investigate the application of multilevel coarsening methods to improve the visualization of large bipartite networks, which are fundamental to SciSci research. These networks, which are often used to model collaborative structures, represent entities such as authors and articles. Using graph coarsening techniques that iteratively reduce network complexity while preserving structural information, the authors present a hierarchical visualization interface. This framework allows users to start with a broad overview and progressively explore finer details by interactively zooming in on selected nodes or subgraphs. The approach offers adaptability by incorporating different strategies and proves effective in revealing structural patterns such as community structures.

Peña-Rocha et al. examine two scholarly document classification systems, comparing a Scopus journal-based assignment method adapted to a fractional model (which assigns weighted categories to references) with an item-by-item reclassification system based on citation origin. The comparative analysis highlights the advantages of the item-by-item system, including a higher percentage of single-category assignments, a more homogeneous distribution of normalized impacts (lower standard deviation), and a better alignment of indicators of excellence across research fields. While the new system does not completely eliminate the differences in discipline size, it improves classification accuracy and provides a more accurate representation of scientific fields. The authors show that this alternative approach offers improvements in bibliometric classification.

Costa and Frigori propose a quantitative methodology that combines Shannon entropy analysis of the words in article titles and fractal dimension calculation of citation networks to assess the temporal evolution of scientific research. By examining entropy fluctuations and structural changes in citation networks of artificial intelligence articles, the study identifies critical phase transitions in research focus characterized by peaks in network connectivity. The results suggest that this approach can be used as a trend-forecasting tool to detect shifts in research trajectories. Although the study focuses on artificial intelligence, the authors mention that the methodology is generalizable and can be applied to other scientific fields.

Lepsch-Cunha et al. analyzed Brazilian research on medicinal plants and herbal medicines, with a focus on the Amazon region and its ethnopharmacological importance. The study used data from Scopus and applied bibliometric and network analysis methods, as well as natural language processing techniques. The authors focus their network analysis on collaboration networks, which include networks of countries and Brazilian institutions. The results show that Brazil is dominant in scientific production related to the Amazon region, mainly from institutions located in the Amazon region. The main research focus includes pharmacology, toxicology, and biochemistry. The study also identifies the technological maturity of Brazilian research, showing a focus on early-stage development and emphasizing the need for increased national and international collaboration. The authors conclude that Brazil, while rich in biodiversity and traditional knowledge, offers significant potential for the global pharmaceutical industry.

Mota et al. systematically mapped the field of Project-Based Learning (PjBL) using scholarly documents from the Web of Science Core Collection. Unlike the other articles on this Research Topic, Mota et al. conducted a literature review. After refining the dataset through specific Web of Science queries, the authors applied network analysis to examine relationships within the research corpus. Their findings highlight that PjBL research is primarily concentrated in the fields of engineering and education, as evidenced by the co-occurrence network of research areas. In addition, the study reveals a steady increase in the adoption of PjBL, supporting the expectation that it will continue to be a key component of global education.

The readers are going to see that the articles on this topic highlight the potential of network science as a powerful tool for understanding various aspects of SciSci, particularly in the context of data analysis [e.g., developing visualization tools (Valejo et al.)] and real-world applications [e.g., introducing novel article-level classification methods (Peña-Rocha et al.)]. This Research Topic opens avenues at the intersection of network science and the science of science, two evolving fields that may yield surprising results as higher-quality data becomes available and methods continue to improve.

## References

[B1] Fortunato S. Bergstrom C. T. Börner K. Evans J. A. Helbing D. Milojević S.. (2018). Science of science. Science 359:eaao0185. 10.1126/science.aao0185 29496846 PMC5949209

